# Current concepts in bone metastasis, contemporary therapeutic strategies and ongoing clinical trials

**DOI:** 10.1186/s13046-017-0578-1

**Published:** 2017-08-11

**Authors:** Andrew S. Gdowski, Amalendu Ranjan, Jamboor K. Vishwanatha

**Affiliations:** Institute for Molecular Medicine, University of North Texas Health Science Center, 3500 Camp Bowie Blvd, Fort Worth, TX 76107 USA

**Keywords:** Bone metastasis, Therapies, Clinical trials

## Abstract

**Background:**

Elucidation of mechanisms regulating bone metastasis has progressed significantly in recent years and this has translated to many new therapeutic options for patients with bone metastatic cancers. However, the rapid rate of progress in both the basic science literature and therapies undergoing clinical trials makes staying abreast with current developments challenging. This review seeks to provide an update on the current state of the science in bone metastasis research and give a snap shot of therapies in clinical trials for bone metastatic cancer.

**Main body:**

Bone metastasis represents a difficult to treat clinical scenario due to pain, increased fracture risk, decreased quality of life and diminished overall survival outcomes. Multiple types of cancer have the specific ability to home to the bone microenvironment and cause metastatic lesions. This osteotropism was first described by Stephen Paget nearly 100 years ago as the ‘seed and soil’ hypothesis. Once cancer cells arrive at the bone they encounter a variety of cells native to the bone microenvironment which contribute to the establishment of bone metastatic lesions. In the first part of this review, the ‘seed and soil’ hypothesis is revisited while emphasizing recent developments in understanding the impact of native bone microenvironment cells on the metastatic process. Next, approved therapies for treating bone metastasis at the systemic level as well as those that target the bone microenvironment are discussed and current National Comprehensive Cancer Network (NCCN) guidelines relating to treatment of bone metastases are summarized. Finally, all open interventional clinical trials for therapies relating to treatment of bone metastasis have been complied and categorized.

**Conclusion:**

Understanding the recent advancements in bone metastasis research is important for continued development of novel bone targeted therapies. The plethora of ongoing clinical trials will hopefully translate into improved treatments options for patients suffering from bone metastatic cancers.

## Background

Treatment options and survival outcomes for patients with many types of cancer have improved during the past 50 years [1, 2]. While these improvements are encouraging, those patients who present with metastatic cancer almost ubiquitously face poor prognosis. Patients with metastatic solid tumors are generally not candidates for surgical resection of their primary tumor which immediately limits therapeutic options. Additionally, there is ample room for improvement in the repertoire of the medical therapeutic options that are currently approved for these patients with metastasis. Understanding the mechanisms and engineering solutions is critical to advancing therapies and improving outcomes in patients who develop metastases. Indeed, new therapeutics are under development and in clinical trials with the goal to improve survival, alleviate pain and decrease fracture risk in patients with bone metastatic cancers.

### “Seed and Soil” hypothesis

**Fig. 1:**
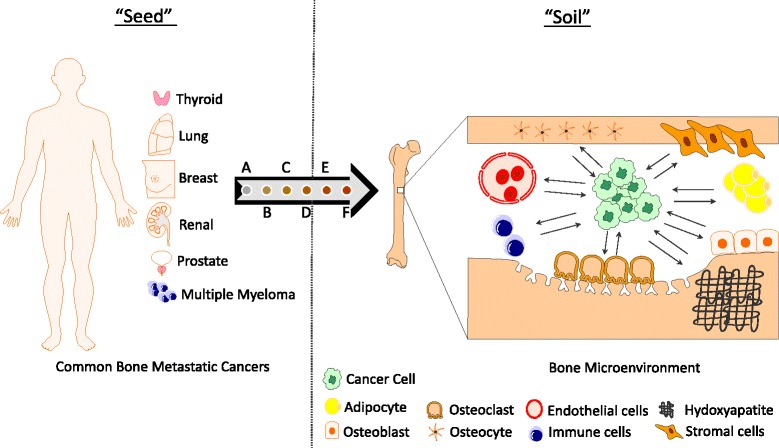
Depiction of the seed and soil hypothesis. The most commonly bone metastatic cancers are thyroid, lung, breast, renal, prostate, and multiple myeloma. The bone microenvironment can be viewed as the soil and contains multiple entities that impact cancer cell survival and establishment of bone lesions. The metastatic process involves: (A) Primary tumor, (B) Angiogenesis, (C) Local invasion and intravasation, (D) Dissemination via circulation, (E) Extravasation, and (F) Colonization of a metastatic site (bone). Components of the bone microenvironment include: endothelial cells, osteocytes, stromal cells, adipose cells, osteoclasts, osteoblasts, T cells, B cells, and the chemical structure of the bone

Tumor cells necessarily require interaction with the microenvironment of a specific host organ to create a metastatic lesion [3]. This concept was first described over 100 years ago by the English surgeon, Stephen Paget. Paget described the ‘seed and soil’ hypothesis in which he sought to explain why certain cancers favored developing metastasis in specific organs. In his research, he studied the autopsy results of patients who had various primary tumors and found that these patients had specific organ patterns where the metastases developed. For example, he found that women who had breast cancer had a much greater probability of having metastases to the bone than any other organ. He explained these results by proposing that the tumor cells acted as ‘seeds’ and have an affinity for particular organs or the ‘soil’. Thus, metastases will develop when the right combination of a compatible seed is planted in the right soil [4, 5] (Fig. 1).

### Metastatic process

This complicated process is precisely coordinated and the molecular basis underlying its orchestration from initiation to development of distant metastasis is a vigorous area of research. The initial step in metastasis necessitates that the cancer cells escape from the primary tumor and into systemic circulation. Cancer cells accomplish this through a process termed epithelial-to-mesenchymal transition (EMT). This transformation enables epithelial type cancer cells to undergo a phenotypic change to exhibit mesenchymal traits such as loss of cell surface intercellular adhesion proteins and loss of epithelial polarization [6]. The cancer cells also secrete extracellular proteolytic enzymes to dissolve the extracellular matrix and escape the physical environment of the tumor stroma [7]. The most prominent of these factors are the matrix metalloproteinase enzymes [8]. After an adequate amount of the extracellular matrix has been dissolved, the cancer cells become locally invasive and begin to migrate into surrounding tissue [9]. Cancer cells continue to migrate through the endothelial cells to gain access to systemic circulation through a process called intravasation [10]. This process is mediated at the vascular level by the tortuous and leaky tumor vasculature [11] as well as cell signaling aberrations in the cancer cells that increase cellular adhesion factors such as integrin B1, enabling the cancer cells to interact with the endothelium [12].

Once cancer cells invade blood vessels and get into systemic circulation, they are termed circulating tumor cells (CTC) and are presented with a new set of challenges. The circulatory system is an inhospitable environment but metastatic tumor cells have mechanisms to improve their chances of survival. [13] One example of how these cells survive is by inhibiting anoikis. Anoikis is normally an apoptotic process which cells undergo when there is loss of cell-matrix or cell-cell interactions. As such, the deregulation of anoikis in the context of metastasis is likely present before cancer cells intravasate and continues during the circulation process [14]. One specific example that has been linked to anoikis resistance is a tyrosine kinase receptor, TrkB. It has been shown that overexpression of this receptor on the membrane of cancer cells, results in activation of the phosphatidylinositol-4,5-bisphosphate 3 kinase (PI3K)-AKT pro-survival pathways [15]. Cancer cells also have mechanisms to escape destruction by immune cells, such as macrophages, by upregulating certain cell surface proteins like CD47 [16].

The two main factors impacting the location CTCs will develop a metastatic lesion are: blood flow and molecular signaling. This is particularly true for cancers that metastasize to the bone. Consider the example of breast cancers which have a preference to metastasize to the thoracic spine due to venous drainage of the breast from the azygos venous system communicating with the plexus of Batson in the thoracic region [17]. This is in comparison to lung cancers which show a more general skeletal distribution due to venous drainage from the pulmonary veins into the left side of the heart and from there dissemination to systemic circulation [18]. Alternatively, the majority of prostate cancer metastasis are seen in the axial skeleton in the lumbar spine, sacrum, and pelvis due to venous drainage of the prostate through the pelvic plexus [19]. Further, colon cancer is known to metastasize to the liver due to portal venous drainage [20]. However, blood flow patterns do not fully explain the distribution of metastatic lesions. In addition to blood flow, a plethora of other factors and signaling events are crucial in the dissemination of CTCs. One well documented process is CTC homing to the bone marrow microenvironment.

One of the signaling pathways regulating CTC homing to the bone is the CXCL12-CXC-chemokine receptor 4 (CXCR4) axis [21]. CXCL12, also called stromal derived factor-1 (SDF-1), is a chemokine factor that is made by bone marrow mesenchymal stem cells, endothelial cells, and osteoblasts. CXCL12 binds primarily to the g-protein coupled receptor, CXCR4, activating several divergent intracellular signaling pathways that are involved in cellular processes including: cell survival, gene transcription, chemotaxis, and expression of integrins such as integrin avB3 on the surface of the CTCs [22]. The increased expression of α_V_β_3_ on the surface of the metastatic prostate tumor cells has been shown to cause it to adhere to endothelial cells of the bone marrow [23]. The CXCL12-CXCR4 axis is not only important for CTC from solid tumors, but also plays a significant role in hematopoietic stem cells and leukemia cells homing to the bone marrow [24, 25]. Other molecules have shown importance in the adhesion process as well. These include other integrins such as α4β1 [26], annexin II [27], and E-cadherin [28].

In addition to the significance of CXCL12-CXCR4 axis for cell adhesion in cancer cells, this signaling pathway has also been shown to be important in cancer cell survival. It has been demonstrated that in breast cancer cells that aberrantly express the non-receptor cytoplasmic tyrosine kinase, Src, have improved survival in the bone marrow. It was shown that Src mediates this improved survival through Akt signaling in response to CXCL12-CXCR4 stimulation and through increasing resistance to TNF-related apoptosis-inducing ligand (TRAIL) specifically in the bone marrow microenvironment [29].

### Bone microenvironment

Once the process of homing and extravasation have taken place, the metastatic cells encounter native bone microenvironment cells. These cells play a vital role in maintaining homeostasis of the bone and include: osteoclast, osteoblasts, osteocytes, endothelial cells, and cells of the bone marrow. The growth and dynamic turnover of bone is regulated through precise signaling between these cells. Alteration in the homeostasis of these native cells can have disastrous effects. When cancer cells Infiltrate the bone, the lesions that develop are traditionally classified as either osteolytic, in which bone is broken down, or osteoblastic, in which bone is formed [30]. These processes are not binary. Rather, both the osteoclastic and the osteoblastic activities are generally activated in all metastatic bone lesions [31]. However, depending on which process is dominant the radiological appearance of a bone metastasis is either lytic, sclerotic, or mixed. The cancers that conventionally cause osteolytic lesions are breast and multiple myeloma [32]. These types of lesions can be particularly dangerous and have the highest rates of fracture. Osteoblastic lesions are seen most often with metastases from prostate cancer [33] and have an increased risk of fracture due to the altered architecture of the bone but not to the same degree in osteolytic lesions.

The cells responsible for bone resorption are known as osteoclasts. These cells are monocyte-macrophage derived multinuclear cells that are initially inactive [34]. Osteoclasts generally are positioned in resorption pits and when activated secrete cathepsin K. This creates an acidic environment on the underside of the osteoclast where the cell maintains a sealed ruffled border [35]. Osteoclast activation is under the control of both systemic factors as well as locally secreted cytokines. Parathryroid hormone, 1,25-dihydroxyvitamin D_3,_ and prostaglandins cause upregulation of receptor activator of nuclear factor-κB ligand (RANKL) [36, 37]. RANKL is a family member of tumor necrosis factors (TNF) which is expressed on the membrane surface of both stromal cells and osteoblasts as well as released by active T cells. Structurally, RANKL is a homotrimeric type II membrane protein with three isoforms. [38] The full length version of RANKL is denoted RANKL1. RANKL2 is shorter due to a portion of the intracytoplasmic domain missing. While RANKL3 is the soluble isoform and has the N-terminal portion deleted [38]. RANKL activates osteoclasts by signaling though its receptor, RANK, with subsequent activation of nuclear factor-κB and Jun N-terminal kinase pathways. Locally, stromal cells and osteoblasts also activate osteoclasts by production of macrophage colony stimulating factor. Additional control over osteoclast activation is managed by osteoprotegerin, which is a decoy receptor for RANKL and is normally present in the marrow [39]. An altered ratio of osteoprotegerin to RANKL can result in osteopetrosis or osteopenia [40, 41].

In addition to the osteoclasts, osteoblasts have a major role in maintaining the bone structure. These cells originate from mesenchymal stem cells and are responsible for synthesizing new bone [42]. This is a critical function, not only during development but also throughout life. Several factors allow for successful differentiation of osteoblasts such as bone morphogenetic proteins (BMPs), platelet-derived growth factor (PDGF), fibroblast growth factor (FGF) and transforming growth factor β (TGF-β) [43, 44]. The differentiation of osteoblasts is not as well understood as the process in osteoclasts, but one factor that is known to drive the differentiation process is the transcription factor Runx-2, also called core-binding factor alpha 1 (CBFA1) [45]. As osteoblasts become more mature they secrete osteocalcin and calcified matrix, eventually becoming osteocytes as they are encapsulated within the bone [46].

Osteocytes make up approximately 90% of the bone cells in the adult human, however less is known about their role in bone metastasis than osteoblasts and osteoclasts [47]. Even though osteocytes are surrounded by the bone matrix, they communicate through an extensive lacunar-cannicular network which connects the osteocytes to other osteocytes, the bone surface, and marrow cells. They regulate osteoclast development through expression of: RANKL, macrophage colony stimulating factor (M-CSF) and osteoprotegerin (OPG). In addition, they can inhibit osteoblasts by expression of sclerostin [48]. Osteocytes have an interesting ability to respond to stress and pressure. In fact, increased pressure in the bone from prostate cancer metastasis can upregulate matrix metalloproteinases and CCL5 in osteocytes resulting in increased tumor growth [49]. IL-11 has been shown to be released from apoptotic osteocytes causing osteoclast differentiation [50]. Additionally, physical interactions and secreted factors from cancer cells such as multiple myeloma cells impact osteocyte function [51].

Endothelial cells comprise another component of the bone microenvironment that contribute to the bone metastatic process through a variety of mechanisms. Endothelial cells in the metaphysis of long bones are known to constitutively express P-selectin, E-selectin, vascular adhesion molecule 1 and intercellular adhesion molecule A which aid in CTC adhesion when they travel through the bone marrow [52]. The physical architecture of the bone vasculature also plays a role in the homing process. The large volume of sinusoids decreases blood flow velocity thus decreasing shear forces and increasing the favorability for attachment of cancer cells [53]. Additional mechanisms by which the endothelial cells promote bone metastatic lesions are through promotion of cell dormancy and neovascularization for metastatic growth [54]. Tumor cells can secrete angiogenetic factors such as vascular endothelial growth factor (VEGF) and IL-8 that can serve to increase survival of the tumor cells and neovascularization [55].

More recent evidence has demonstrated the importance of immune cells in the development of bone metastases. The bone marrow is a major reservoir for dendritic cells, macrophages, myeloid derived cells, and different subsets of T cells [56]. T cells have been shown to regulate bone resorption in both solid tumors bone metastasis and multiple myeloma [57, 58]. T cells and B cells also produce RANKL and can impact osteoclastogenesis. IL-7 is an important cytokine that mediates interactions between T cells and the proliferative bone metastatic environment [59]. Myeloid derived suppressor cells from the bone marrow have proven to be impactful in their ability to drive cancer progression through suppression of innate and adaptive immune responses, impairing T cell antigen recognition and promotion of T regulatory cells [60–62]. In the microenvironment of multiple myeloma patients, dendritic cells and IL-6, IL-23 and IL-1 are involved in increased Th17 cells, which increase IL-17 and can promote osteoclast and myeloma proliferation [48]. Additionally, IL-17 has been shown to be a growth factor for both prostate and breast cancer cells [63, 64].

During development, the bone marrow changes from being predominately red or hematopoietic marrow and having very little adipocytes or yellow marrow to being composed of approximately 70% adipose tissue, by the age of twenty five [65]. These adipocytes were previously thought to be inert but now are considered to have a significant impact on the development of bone metastasis in the microenvironment. It has been proposed that adipocytes play a supporting role for cancer cell survival in the bone marrow as an energy source [66, 67]. Bone marrow adipocytes also secrete several pro-inflammatory mediators such as IL-1B, IL-6, leptin, adiponectin, vascular cell adhesion molecule 1 (VCAM-1), tumor necrosis factor alpha (TNF-alpha) and CXCL12 that increase bone tropism, proliferation, and survival of certain cancer cells [65, 68–70].

Additionally, cancers cells that are already within the bone microenvironment play in impactful role on the further development of these metastatic lesions. Important activating factors expressed by the prostate cancer cells that create bone metastasis include: FGFs [71] and BMPs [72]. It has been shown that FGF can act through autocrine or paracrine signaling [73]. Binding of FGF to an FGF receptor results in activation of multiple signal transduction pathways beneficial for the tumor. These stimulated pathways include: phosphatidylinositol 3-kinase (PI3K), phospholipase Cγ (PLCγ), mitogen-activated protein kinase (MAPK), and signal transducers and activators of transcription (STAT) [31, 73]. The resulting stimulation of these pathways from multiple FGFs results in simulation of the cells in the bone microenvironment and the cancer cells during metastatic lesion development [31].

The mineral structure of the bone itself presents additional components that can serve to enhance bone metastatic lesions. Encased within the hydroxyapatite are a number of factors such as: bone morphogenetic proteins, insulin like growth factors I and II, platelet-derived growth factor, transforming growth factor-beta and fibroblast growth factor [74]. These factors become important when liberated from the mineralized hydroxyapatite by promoting growth and proliferative effects on tumor cells and worsening the metastatic lesion.

## Bone metastases therapies

### Introduction to treatment concepts

Therapeutic strategies for bone metastatic cancers rely on three main principles: 1.) The cancer cells should be treated. This is critical because the cancer cells are the initial insult which cause bone metastatic lesions to develop. If cancer cells continue to proliferate and divide, it should not be expected that survival time will be extended. This principle can be broken down further into therapies that are cytotoxic and kill the cells, hormonal deprivation, or targeted agents that inhibit specific signaling pathways; 2.) Targeting the bone microenvironment is impactful. As was discussed in the above sections on the bone microenvironment, the complex biological signaling between cancer cells and bone resident cells creates a vicious cycle. Disruption of these interactions represents a therapeutic opportunity; 3.) Palliative therapies focus on alleviating symptoms associated with bone metastasis. This becomes an area that can be very impactful on the quality of life for these cancer patients as bone metastasis can be extremely debilitating and painful.

Most of the following discussion on approved therapeutics will focus on prostate, breast, and multiple myeloma. These are the most common cancers which cause bone metastatic lesions and thus represent the bulk of research efforts to understand the mechanisms involved. Patients with other cancers such as kidney, thyroid, lung and melanoma can also present with metastasis to the bone. There are many treatment commonalities between the various cancers that metastasize to the bone and strategies appropriate for one type of cancer are often effective for others.

### Approved therapeutic agents

#### Bisphosphonates

Bisphosphonates are a unique drug class that have been used in multiple clinical settings for their ability to prevent bone loss. In addition to their role in the treatment of patients with bone metastatic cancer, they are also clinically effective for use in osteoporosis, Paget’s disease and osteogenesis imperfecta [75–77]. However, use of these agents is not without the potential for adverse side effects such as osteonecrosis of the jaw, esophageal irritation, and fractures [78, 79].

The bone targeting ability of bisphosphonates for the mineral structure of hydroxyapatite is due to their chemical configuration. Bisphosphonates consist of two phosphonate groups that are bound by a carbon atom. Additional functional groups have been attached to the central carbon atom which confers different pharmacological properties to these molecules. The two phosphonate groups in these drugs allow high binding affinity to the hydroxyapatite structure and this is enhanced in areas of high bone turnover such as bone metastatic lesions [80, 81]. Depending on the side groups of the bisphosphonate molecule either a bidentate bond forms through calcium ion chelation on the surface of the hydroxyapatite by a stronger tridentate bond can form. [82, 83]

Bisphosphonates can be subdivided based on the presence of a nitrogen containing side group. The clinically approved nitrogen containing molecules are ibantdronate, pamidronate, alendronate, risedronate and zoledronate. The nitrogen free bisphosphonates are clodronate, tiludronate and etidronate [84]. Zoledronic acid has been shown to have the best efficacy among the bisphosphonate molecules and was approved based on its ability to prolong the time to symptomatic skeletal related events but did not show an improvement in overall median survival when compared to the placebo [85].

The overall mechanism of bisphosphonates is to inhibit bone resorption through its apoptotic effects on osteoclasts after being endocytosed. Uptake causes osteoclast apoptosis through one of two main mechanisms depending on the class of bisphosphonate. Endocytosis of non-aminobisphosphonates results in disruption of ATP supply as osteoclasts metabolize this class into analogues of ATP and eventually undergo apoptosis [86]. The mechanism by which amino-bisphosphonates cause apoptosis in osteoclasts is through inhibition of farnesyl pyrophosphate synthase and the mevalonate pathway [87]. Additionally, osteoclast apoptosis limits the vicious cycle of signaling that takes place between the osteoclasts and cancer cells in the bone microenvironment.

#### Denosumab

Denosumab was FDA approved based on the study by Fizazi et al. in 2011 where they showed a prolonged time to skeletal related event by 3.6 months compared to zoledronic acid [88]. Denosumab is a human monoclonal IgG2 antibody that acts by binding to both membrane bound and soluble RANKL with high affinity [89, 90]. As was discussed in earlier sections, RANKL is a molecule that is primarily secreted by osteoblasts and upon attachment to RANK (located on osteoclasts) stimulates osteoclastic activity. The exact location of binding of denosumab is on the DE loop region of RANKL, which forms a contact with RANK [91]. Thus, treatment with denosumab prevents this contact and inhibits bone resorption. In addition to the RANKL that is secreted by osteoblasts, inflammatory cells and stromal cells also secrete RANKL and impact tumor development [92, 93]. In the clinical setting, denosumab has shown positive results in preventing pain [94, 95], lessening hypercalcemia of malignancy [89, 96] and may also have effects on tumor cells independent of its role in bone homeostatsis [89].

#### Radioisotopes

Radioisotopes also play a role in the treatment of bone metastasis. Ideal candidates for this type of therapy are generally those with osteoblastic or mixed metastatic lesions that are multifocal and causing significant pain [97]. Approved radioisotopes for treating bone metastasis are either members of the alkaline earth metals or conjugated to ligands that can direct the radioisotope to the bone. Alkaline earth metals have the same electron valence as calcium so they are concentrated to areas of high bone turnover along with calcium. As a class, these agents are effective at reducing pain associated with bone metastasis but haven’t shown to be effective at prolonging overall survival until the most recently approved radioisotope, radium-223 [98, 99].

Clinically approved radioisotopes can be divided into β-emitters and α-emitters. Two β-emitters, Stontium-89 and Samarium-153, are approved for treating bone pain in patients with bone metastases. These agents deliver ionizing radiation and incorporate into the bone. Strontium can incorporate due to its similarity to calcium and Samarium-153 has been conjugated to ethylenediaminetetramethylene phosphate (EDTMP) which can chelate calcium to allow it to home to the bone [100]. These β-emitters are considered outdated due to other therapeutics with stronger evidence [101].

Radium-223 is an α-alpha emitting radioisotope. It has been approved based on the results of the ALSYMPCA trial after demonstrating not only prolonged time to skeletal related event by 5.8 months as compared to a placebo but also increased overall median survival by 3.6 months [102]. Alpha-emitters can deliver high radiation but the depth of radiation penetration in tissues is less, making them more targeted [103]. As a group, radiopharmaceuticals that target the bone have high rates of myelosuppresion [104]. The adverse effects of Radium-223 appear to be less, with only mild thrombocytopenia [105].

#### Hormonal therapy and chemotherapy

One of the most important goals in the treatment of bone metastatic cancer is disease control. If a cancer is localized, surgery or radiation therapy are generally the first choice. However, for advanced bone metastasis disease, systemic therapy is often required with either cytotoxic agents, targeted therapies, hormonal therapy or a combination of the above. In advanced hormonally driven tumors such as prostate and breast, the first line treatment is hormone deprivation to cut off the proliferative signaling in the cancers. The standard treatment for men with advanced prostate cancer for the past 70 years has been androgen deprivation therapy [106, 107]. There is typically a good initial response to treatment but almost inevitably the patient will become refractory to the treatment and will progress to castration resistant prostate cancer in a period of 18 to 24 months [108]. As the cancer progresses, it will metastasize to the bone in 90% of patients [109] and at this point overall survival is generally less than 2 years [110].

Two newer anti-androgen agents are approved in the setting of castration resistant bone metastatic prostate cancer. Abiraterone inhibits 17-α-hydroxylase/17,20 lyase, which is a testosterone synthesis enzyme that is found in the adrenals, testes and tumor [111]. Enzulatamide is an antiandrogen and exerts its effect by inhibiting nuclear translocation of the androgen receptor, inhibiting the androgen receptor from binding to DNA and blocking co-activator recruitment [101, 112]. The androgen receptor also promotes growth in the bone microenvironment through its expression and activity in the bone microenvironment stromal cells [113].

Cytotoxic chemotherapy is also approved in the context of bone metastatic prostate cancer. Docetaxel is a microtubule inhibitor and was the first chemotherapeutic to show a survival benefit in these patients [114]. More recent results of the STAMPEDE trial showed a survival benefit in prostate cancer patients when docetaxel was started earlier in the treatment course along with long term androgen deprivation treatment [115]. Cabazitaxel is a newer generation taxol and was developed to treat patients who have previously been treated with docetaxel. It has been chemically modified in two locations from the previous docetaxel drug. These alterations give it decreased affinity for the P-glycoprotein pump which on many advanced cancer cells can pump chemotherapy out of the cell rendering it resistant to therapy. It was approved based on the results of the TROPIC trial which showed an overall survival benefit compared to mitoxantrone in patients who were previously treated with docetaxel [116].

The concepts that guide the standard of care for patients with bone metastatic breast cancer are similar to those guiding prostate cancer therapy. Treatment options include systemic agents against the cancer, bone-targeted agents and local therapy as well [117]. The current recommendation is for initiation of endocrine therapy in women who experience recurrence and who are estrogen receptor positive, with the exception if there is rapidly developing disease and organ involvement, in which case chemotherapy should be offered [118]. In addition, bone targeted agents such as bisphosphonates and denosumab are important in delaying skeletally related events such as fractures and for improvement in pain.

#### Immunotherapy

Development and approval of immunotherapy for cancers in general has made considerable progress and attracted interest in recent years. In the advanced prostate cancer field, Sipuleucel-T has been approved after showing a survival benefit in castration-resistant prostate cancer patients who are asymptomatic or minimally symptomatic [119]. It is made using a patient’s own mononuclear cells that are sent to a central processing facility and treated with prostatic acid phosphatase and granulocyte/macrophage colony stimulating factor. These cells are injected back into the patient and the antigen presenting cells activate the patient’s T cells to attack the prostate cancer [120]. As the field of immune-oncology continues to expand, specific bone directed therapies may materialize.

### Other treatment modalities

#### Percutaneous minimally invasive techniques

Treatments such as percutaneous vertebroplasty, kyphoplasty, and radiofrequency ablation are often employed as a palliative measure in the treatment of patients with bone metastatic spinal tumors [121]. In the percutaneous vertebroplasty procedure bone needles are placed into the vertebral body, and polymethylmethacrylate (quick setting bone cement) is injected. The reduction in pain is likely due to restoration of vertebral height and the exothermic nature of the bone cement as it sets [121]. Balloon kyphoplasty is like vertebroplasty but utilizes a balloon to control bone cement extravasation in the spine [122]. Radiofrequency ablation uses alternating current to generate heat and multiple mechanisms may be contributing to reduction in pain such as: cancer cell death causing reduction in pain inducing cytokines, decreasing size of cancer bone lesions, destruction of pain fibers and inhibiting osteoclastogenesis [123]. The goal of these therapies is palliation of pain symptoms so that overall quality of life is improved.

#### Radiation therapy

Radiation therapy is another palliative approach to treating bone metastasis. It is a non-invasive and effective way to improve pain from these lesions generally within 2–6 week of treatment [117]. This treatment can be performed by dose fractionation in which multiple doses of radiation are given or administered in a single-dose [124–126]. The ideal candidates for this therapy are those with solitary or oligometastatic disease to the bone [127].

#### Surgery

Surgical intervention is generally not the first option in patients with bone metastasis but may be helpful in certain instances. For spinal tumors, hormonal and radiation treatments are considered first. However, decompression laminectomy and fixation as well as en bloc spondylectomy may be beneficial in appropriately selected patients [128]. Treatments for metastasis to long bones include internal fixation, external fixation and prosthesis placement [129, 130].

#### NCCN guidelines summary of treatment of bone metastatic cancers

Table 1 is a compilation of the individual 2017 National Comprehensive Cancer Network (NCCN) cancer treatment guidelines for recommendations on treating bone metastasis. Cancers with the highest bone metastases prevalence were selected.

### Current clinical trials in bone metastasis

A review of current, open, interventional clinical trials for “bone metastasis” was performed using the clinical trials database at clinicaltrails.gov and 445 trials were found. Relevant clinical trials on cancers involving prostate, breast, renal, thyroid, lung, multiple myeloma, or trials involving therapies for multiple types of cancers were included. This information is included in Table 2.

**Table 1:** Treatment options for various types of bone metastatic cancers

	Prostate	Breast	Renal	Lung	Thyroid	Multiple Myeloma
Systemic Therapy	Yes	Yes	Yes	Yes	Yes	Yes
Bone- Targeted	Denosumab	Denosumab	Denosumab	Consider: Denosumab	Denosumab	Pamidronate
	Zoledronic Acid	Zoledronic Acid	Zoledronic Acid	Zoledronic Acid	Pamidronate	Zoledronic Acid
	Radium-223	Pamidronate			Zoledronic Acid	
Radiation Therapy	Yes	Yes	Yes	Yes	Yes	Yes
Vitamins	Calcium Vitamin D	Calcium Vitamin D	Calcium Vitamin D	Not Mentioned	Not mentioned	Not mentioned
Notes	Possible use of Sr-89 or Sm-153				Consider embolization prior to surgical resection to reduce hemorrhage

**Table 2:** Summary of Current Clinical Trials for Bone Metastatic Cancers

Therapy	Clinical Trial Description	Multiple Cancers	Prostate	Breast	Renal	Thyroid	Lung	Myeloma
Radiotherapy	Hypofractionated radiotherapy regimen	NCT02376322						
	SBRT vs EBRT	NCT00922974						
	1 vs 2 fractions of EBRT	NCT02699697						
	Dose regimen of radiotherapy	NCT02163226						
	SBRT + ADT	NCT02563691						
	IMRT vs EBRT	NCT02832830						
	EBRT +/− hyperthermia	NCT01842048						
	Surgery +/− postoperative radiotherapy	NCT02705183						
	Treatment of opiod refractory pain with pituitary radiosurgery	NCT02637479						
	LHRH agonist + Enzulutamide +/− SBRT		NCT02685397					
	ADT +/− radiotherapy		NCT02913859					
	SBRT + anti PD-1 antibody			NCT02303366				
	SBRT with sunitinib				NCT02019576			
	FDG-PET guided radiotherapy with conventional dose vs FDG-PET with SBRT dose escalation	NCT01429493						
	Conventional radiotherapy vs SBRT	NCT02364115						
	SBRT workflow	NCT02145286						
	SBRT	NCT02880319						
	Single fraction SSRTvs multple fraction SSRT	NCT02608866						
	Zoledronic acid + high dose radiotherapy or low dose radiotherapy						NCT02480634	
	Conventional radiotherapy vs SBRT	NCT02512965						
	Observation vs SBRT vs SBRT +18F–DCFPyL		NCT02680587					
Small Molecule	Zoledronic acid (prevention)						NCT02622607	NCT02286830
	Docetaxel + zoledronic acid +/− apatinib						NCT03127319	
	Dose escalation of sirolimus + cyclophosphamide, methotrexate, zolendronic acid	NCT02517918						
	Dosing scheducle of pamidronate or denosumab or zolendronate	NCT02721433						
	Zoledronic acid or densoumab +/− amorphous calcium carbonate		NCT02864784					
	Calcifediol + denosumab or zoledronic acid	NCT02274623						
	Cabozantinib		NCT01703065					
	Receptor tyrosine kinase inhibitor	NCT02219711						
	Selinexor (selective inhibitor of nuclear export)		NCT02215161					
	Docetaxel + clarithromycin vs cabazitaxel + clarithromycin		NCT03043989					
	Enzalutamide + LHRH analogue therapy vs bicalutamide + LHRH analogue therapy		NCT02058706					
	Copper + disulfram		NCT02963051					
	Palbociclib + tamoxifen			NCT02668666				
	Denosumab + enzalutamide +/− abiraterone and prednisone		NCT02758132					
Mab	Dosing schedule of denosumab	NCT02051218						
	Denosumab in patients with circulating tumor cells plus bone metastasis			NCT03070002				
	Denosumab + hormonal therapy			NCT01952054				
	Pembrolizumab		NCT02787005					
	Radium 223 +/− pembrolizumab		NCT03093428					
Radioisotopes	ADT +/− radium 223		NCT02582749					
	Hormonal therapy +/− radium 223			NCT02258464				
	EBRT +/− radium 223		NCT02484339					
	Radium 223		NCT03062254			NCT02390934	NCT02283749	
			NCT03002220					
			NCT02312960					
Cellular therapy	Genetically modified dendritic cells + cytokine induced killer cells						NCT02688686	
	Sipuleucel-T +/− radiation therapy		NCT01833208					
	Engineered autologous T cells + cyclophosphamide		NCT01140373					
	Dendritic cell based cryoimmunotherapy + cyclophosphamide +/− ipilimumab		NCT02423928					
	Sipuleucell-T +/− radium 223		NCT02463799					
HIFU	HIFU vs EBRT	NCT01091883						
	MRI guided HIFU	NCT03106675						
		NCT02718404						
		NCT00981578						
		NCT01833806						
		NCT02616016						
Surgery	Surgery +/− radiation therapy	NCT01428895						
	Radical Prostatectomy + ADT		NCT02454543					
	Intraoperative radiotherapy with kyphoplasty		NCT02480036					
	Kyphoplasty vs vertebroplasty	NCT02700308						
	Resection of primary breast tumor in stage IV patients			NCT02125630				
Other	Modified Polio virus		NCT03071328					
	Somatostatin		NCT02631616					
	Intermittant Fasting		NCT02710721					
	Bifunctional macromolecular poly-bisphosphonate		NCT02825628					
	Thermal ablation + steriotactic radiosurgery	NCT02713269						
	Pembrolizumab + pTVG-HP plasmid DNA vaccine		NCT02499835					
	Isometric resistance training	NCT02847754						
	Fentanyl transmucosal	NCT02426697						
	Fentynl intranasal	NCT03071744						
	Tanezumab (for pain)	NCT02609828						
	QOL with denosumab or bisphosphonates	NCT02839291						
	Cryoablation	NCT02511678						

## Conclusions

Research into the molecular mechanisms of metastatic cancer, particularly bone metastatic cancer, has progressed rapidly in the past decade. Understanding the interactions and signaling processes at the bone microenvironment level has proven beneficial in advancing the field. Indeed, this knowledge has translated into the development and subsequent approval of several new targeted agents for patients with bone metastatic cancers. There are many promising therapeutic options in current pre-clinical development and in clinical trials that give hope for improved treatments and outcomes in patients with bone metastatic cancer.

## Abbreviations

ADT: Androgen deprivation therapy
BMPs: Bone morphogenetic proteins
CBFA1: Core-binding factor alpha 1
CTC: Circulating tumor cell
CXCR4: CXCL12-CXC-chemokine receptor 4
EBRT: External beam radiation therapy
EDTMP: Ethylenediaminetetramethylene phosphate
EMT: Epithelial-to-mesenchymal
FGF: Fibroblast growth factor
HIFU: High intensity focused ultrasound
IMRT: Intensity modulated radiation therapy
M-CSF: Macrophage colony stimulating factor
NCCN: National Comprehensive Cancer Network
OPG: Osteoprotegerin
PDGF: Platelet-derived growth factor
PI3K: Phosphatidylinositol-4,5-bisphosphate 3 kinase
QOL: Quality of life
RANKL: Receptor activator of nuclear factor-κB ligand
SBRT: Stereotactic body radiation therapy
SDF-1: Stromal derived factor-1
SSRT: Spinal stereotactic radiation therapy
TNF alpha: Tumor necrosis factor alpha
TNF: Tumor necrosis factors
VCAM-1: Vascular cell adhesion molecule 1
VEGF: Vascular endothelial growth factor
